# Stressed: The Unfolded Protein Response in T Cell Development, Activation, and Function

**DOI:** 10.3390/ijms20071792

**Published:** 2019-04-11

**Authors:** Kyeorda Kemp, Cody Poe

**Affiliations:** 1 Department of Foundational Medical Studies, Oakland University William Beaumont School of Medicine, 586 Pioneer Drive, Rochester, MI 48309, USA; 2 Department of Natural Sciences, Northeastern State University, 3100 New Orleans Street, Broken Arrow, OK 74014, USA; codypoe1@gmail.com

**Keywords:** ER stress, UPR, protein folding, T cells

## Abstract

The unfolded protein response (UPR) is a highly conserved pathway that allows cells to respond to stress in the endoplasmic reticulum caused by an accumulation of misfolded and unfolded protein. This is of great importance to secretory cells because, in order for proteins to traffic from the endoplasmic reticulum (ER), they need to be folded appropriately. While a wealth of literature has implicated UPR in immune responses, less attention has been given to the role of UPR in T cell development and function. This review discusses the importance of UPR in T cell development, homeostasis, activation, and effector functions. We also speculate about how UPR may be manipulated in T cells to ameliorate pathologies.

## 1. Introduction

An important stop on the journey of a secreted protein to the plasma membrane is entry into the endoplasmic reticulum (ER). Newly synthesized peptide sequences that emerge from the ribosome containing an ER signal sequence are bound by a signal recognition protein (SRP). SRP binding slows translation, and the SRP targets the ribosome-nascent chain complex to the protein translocation channel (translocon) in the ER [1]. Upon docking of the ribosome with the ER, the peptide enters the translocon, translation resumes, and the signal sequence is cleaved off.

Newly translated proteins are modified to allow for confirmation of appropriate protein formation in the ER lumen. If protein folding does not proceed appropriately, chaperone proteins will bind to the misfolded protein and retain it within the ER. Retention of the misfolded protein for an extended period activates the endoplasmic reticulum-associated degradation (ERAD) pathway that results in the ubiquitination and eventual degradation of the protein. The rate of protein trafficking is dependent in part on this quality control process. Under normal conditions, ERAD is able to maintain ER homeostasis, but should the cell become overwhelmed with misfolded protein, it must resolve this issue or undergo apoptosis [2,3].

**Figure 1:**
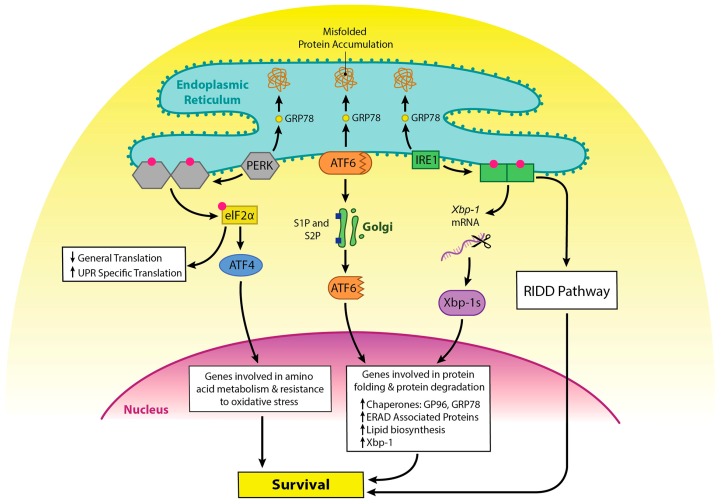
The role of unfolded protein response (UPR) in cell survival upon induction of ER stress. GRP78 is normally associated with PERK, IRE1, and ATF6. GRP78 disassociates from these molecules and binds to unfolded proteins as they accumulate, keeping them sequestered in the ER. Recruitment of GRP78 away from these molecules leads to their activation. PERK dimerizes and auto-phosphorylates upon removal of GRP78. It then phosphorylates eIF2α, which leads to inhibition of translation for most proteins, while UPR specific translation increases. One of those molecules upregulated is ATF4, which functions as a transcription factor and promotes the expression of proteins important in stress response. Upon release of GRP78, ATF6 travels to the Golgi where it is cleaved by S1P and S2P, resulting in a fragment that is active in promoting gene transcription. IRE1 dimerizes and auto phosphorylates as well upon removal of GRP78. It then can splice *Xbp-1* mRNA, allowing for the production of a transcription factor that works in tandem with ATF6 to promote genes involved in protein folding and degradation. IRE1 activates the regulated IRE1-dependent decay (RIDD) pathway which results in the degradation of mRNAs, which reduces the load in the ER. All of these pathways promote cell survival.

**Figure 2:**
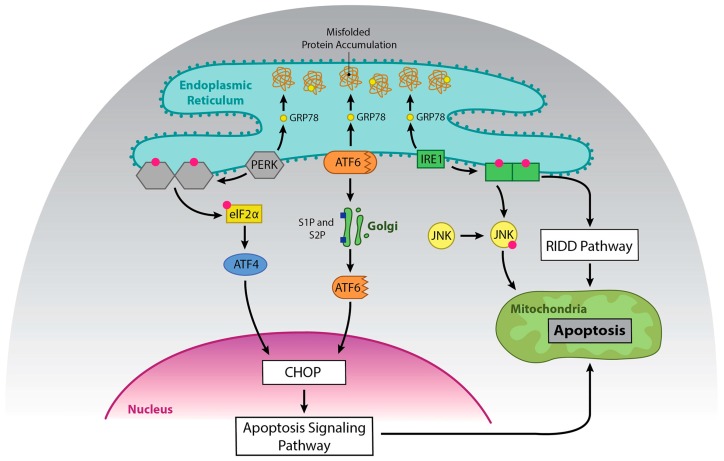
The role of UPR in cell death upon prolonged ER stress. Prolonged activation of UPR can promote apoptosis. Dimerization and phosphorylation of PERK promotes ATF4, which activates CHOP and subsequently apoptosis. ATF6 can also promote upregulation of CHOP. IRE1α can promote apoptosis via activation of JNK and via degradation of pro-survival RNAs by RIDD.

Integrated stress responses (ISR) are general stress-response mechanisms that are evolutionarily conserved amongst eukaryotes [4]. These responses maintain cell homeostasis and are activated by a number of mechanisms such as amino acid depletion, UV irradiation, lipid exposure, viral infection, heme deprivation, oxidative stress, hypoxia, and ER stress due to the accumulation of misfolded protein in the ER [5]. All mechanisms that activate ISR result in a block in general protein translation due to phosphorylation of eukaryotic translation initiation factor alpha (eIF2α) at Ser51, and this helps reduce stress in the ER. The unfolded protein response (UPR) overlaps with the ISR, as stress in the ER activates both responses, and both responses result in phosphorylation of eIF2α [4,6]. However, in the case of ISR, multiple proteins can mediate phosphorylation of eIF2α, including protein kinase RNA-like endoplasmic reticulum kinase (PERK), while UPR mediates phosphorylation of eIF2α through PERK alone. PERK and the other activators of UPR are kept in an inactive state by GRP78, and GRP78 disassociates and binds to unfolded protein, which leads to activation of these proteins [7]. Other molecules are involved in this process [8]; however, they will not be discussed here, as it is beyond the scope of this review. eIF2α phosphorylation also selectively promotes the translation of UPR specific mRNAs, such as activating transcription factor 4 (ATF4). ATF4 increases transcription of genes involved in resolving ER stress and inducing apoptosis (Figure 1 and Figure 2) [9,10]. ATF4 also promotes the expression of *IRE1α* mRNA in mouse cells, and IRE1α is important in UPR [11].

There are two forms of the serine/threonine kinase IRE1. Mucosal tissue contains the isoform IRE1β, while other tissues possess IRE1α [12,13]. IRE1 splices a 26-nucleotide intron from *X box binding protein 1 (XBP1)* mRNA, creating a protein that acts as a transcription factor for UPR-related genes [14]. IRE1 can also regulate a subset of other mRNAs through a process called regulated IRE1-dependent decay (RIDD) [15]. In RIDD, IRE1 preferentially targets and cleaves ER-localized mRNAs at a *Xbp-1 like* consensus site [16,17]. RIDD activity increases under ER stress, and excessive IRE1 activation induces cell death by repressing anti-apoptotic pre-microRNAs; however, it appears to be necessary for normal cell homeostasis [16,18,19]. IRE1 also promotes apoptosis via a pathway that involves Traf2 and JNK [20].

ATF6 is bound to the ER membrane, but when protein homeostasis is disrupted, as in ER stress, GRP78 is released from the luminal domain [21]. ATF6 then migrates to the Golgi apparatus to undergo cleavage, first by Site 1 Protease (S1P) and then by Site 2 Protease (S2P) [22]. This process unmasks the cytosolic domain of ATF6, and ATF6 enters the nucleus where it binds to the ER stress response element (ERSE) containing sequence to activate genes that encode molecules involved in the UPR, ER chaperones, ERAD components, and Xbp-1 [14]. ATF6 and Xbp-1 can work in tandem to promote expression of the above proteins [23]. While ATF6 promotes cell survival, it can promote apoptosis via upregulation of CHOP [24,25,26] (Figure 2).

It is critical that cellular homeostasis is maintained, as failure to do so results in the development of a diseased state in the host, and both UPR and ISR work to maintain homeostasis. The UPR re-establishes normal function in the ER by promoting changes that resolve the accumulation of unfolded protein. Activation of the UPR promotes degradation of terminally misfolded proteins via ubiquitination and the proteasome and attenuated translation of non-essential proteins, while increasing the expression of those that promote protein folding and degradation [7].

Upon antigen recognition, immune cells undergo proliferation and develop effector functions that result in the influx of proteins into the ER, potentially initiating ER stress. There is a wealth of evidence that indicates that ER stress is a major contributor to disease and inflammation [27,28]. More recently, ER stress, ISR, and UPR have been shown to play a role in T cell development and response. This review focuses on the role of ER stress and UPR in T cell development, homeostasis, activation, proliferation, and effector functions. However, molecules that play a dual role in ISR and ER stress will also be highlighted. In addition, we explore how UPR may be manipulated in T cells to protect against cancer, asthma, and rheumatic diseases.

## 2. UPR and T Cell Lineages

T cell development and differentiation results in an increase in protein translation, and the UPR is important in promoting the health of cells that secrete proteins. Moreover, the UPR has been recognized for its important role in regulating a variety of immune cell functions [29,30,31,32]. Increasingly, the UPR is shown to promote T cell survival and gain of effector functions due to its critical role in fostering protein secretion and ER homeostasis in T cells.

### 2.1. The UPR and T Cell Development

While UPR and ER stress are activated during T cell development, it is unknown if they play a direct role. IRE1 is active at the CD4^+^CD8^+^ double positive (DP) stage of T cell development and is downregulated upon the maturation of CD4^+^ T cells [33]. While this hints at a role for UPR in T cell development, it does not provide definitive proof. Amantini et al. showed that activation of the ion channel transient receptor potential cation channel subfamily V member 1 (TRPV1) induces ER stress in mouse thymocytes and implicated TRPV1 in the regulation of UPR molecules [34]. They showed that knocking out TPRV1 downregulated the expression of GRP78 and heat shock protein glucose-regulated protein (GP96, also known as GRP94), and this led to a modest increase in the protein expression of ATF4 and endoplasmic reticulum resident protein 57. Loss of TRPV1 also resulted in increased apoptosis and reduced thymocytes, as well as defects in positive selection, leading to reduced DP thymocytes. While it is tempting to assume that the increased apoptosis is due to the elevated ATF4, TRPV1 is a component of the T cell receptor (TCR) signaling complex [35]. TRPV1 is phosphorylated by the proximal signaling molecule lymphocyte kinase or Lck, assumedly to mediate signaling events downstream of the TCR, as *TRPV1*^−/−^ CD4^+^ T cells have reduced activation of signaling molecules downstream of the TCR. Failure to signal appropriately during thymocyte selection results in cell death, and Lck is critical in this process [36,37]. *ATF4*^−/−^ mouse HSC injected into donor animals are able to develop into T lymphocytes [38]. Therefore, the increased apoptosis found in *TRPV1*^−/−^ thymocytes may be occurring independent of ER stress and UPR.

GP96 appears necessary for T cell development. GP96 is a molecular chaperone critical in the folding and transport of toll-like receptors and integrins, and without GP96 these molecules fail to be transported to their post-ER compartments [39,40,41]. Thymocyte development requires integrin mediated cell-to-cell interactions [42]. GP96 is required for the expression of most integrins on the cell surface of hematopoietic stem cells (HSC) and T cell development, as the absence of GP96 in mouse hematopoietic stem cells (HSC) led to a CD4^−^CD8^−^ double negative (DN)1 to DN2 transitional block [39].

Loss of ribosomal protein 22 (Rpl22) causes a block in αβ cell development, but not γδ T cell development in mice [43]. This defect is due to elevated ER stress caused by an inability to inhibit protein synthesis, which leads to the upregulation of cell cycle regulator and tumor suppressor p53 and the induction of apoptosis [44]. Knocking down PERK in these cells rescues Rpl22 deficient mouse T cells. These results indicate that PERK may play a role in T cell development, albeit in an ATF4 independent manner.

Regulating mitochondrial function is important for T cell development. For example, RhoA deficient mice have reduced thymocytes and increased apoptosis in thymocyte populations, conceivably due to their thymocytes having elevated reactive oxygen species and mitochondrial function [45]. PERK protects the mitochondria during early stages of ER stress by inducing mitochondrial remodeling in an ATF4 independent manner [46]. It is conceivable that PERK may promote T cell development in the thymus in an ATF4 independent manner through its roles in promoting mitochondrial health.

Interestingly, the Rpl22 deficient mice have a block at the DN3 stage of T development. At this stage T cells must signal through the pre-TCR to allow for β-chain selection and progression to the DP CD4^+^CD8^+^ stage [47,48]. DN3 T cells undergo robust proliferation upon β chain selection [49], and it is possible that UPR promotes survival upon ER stress caused by the proliferation. Alternatively, UPR could be important for β chain selection itself. Rpl22 is upregulated by Myc interacting zinc finger protein (M1Z1) in T cells undergoing VDJ recombination [50]. MIZ1 is crucial for lymphocyte development and promotes pre-TCR expression and β chain selection, and loss of MIZ1 or Rpl22 at this stage results in increased p53 mediated apoptosis [43,51,52]. UPR is known to be activated in lymphocyte progenitor stages, and plays an important role in heavy chain rearrangement in B cells, a process that is analogous to β chain rearrangement in T cells [53].

### 2.2. T Cell Activation and Homeostasis

TCR signaling activates UPR in mouse cells, indicating that UPR may play an important role in T cell activation upon antigen stimulation [54,55]. IL-2 induced by TCR stimulation in CD8^+^ T cells correlates with the expression of UPR response molecules such as GRP78, and GRP78 heterozygous mouse CD8^+^ T cells have a defect in proliferation which is restored by exogenous IL-2 [54,56]. Thaxton et al. show that ER stress activated by T cell stimulation plays an important role in the activation of CD4^+^ murine T cells [57]. While IRE1α is upregulated upon TCR stimulation, IRE1α deficient mouse T cells appear to proliferate and produce cytokines upon TCR and CD28 stimulation [55], indicating that IRE1α is not required for T cell activation. ATF6 may also play a role in the regulation of T cell activation. Loss of GP96 leads to maintenance of a naïve state in mouse CD4^+^ T cells [57], and GP96 expression is regulated by ATF6 [58]. Future studies will have to explore if ATF6 directly plays a role in T cell activation.

ER stress and UPR also appears to play a role in T cell homeostasis and proliferation. KDEL receptor 1 (KDELR) is responsible for retrograde transport of ER resident proteins to the ER from the cis-Golgi. Studies using a cell line stably expressing mutant KDELR showed that the inability of KDELR to retrieve molecules to the ER resulted in the upregulation of UPR [59]. T cells in mice with a KDELR mutation have increased eIF2α phosphorylation and upregulation of CHOP, leading to a reduction of naïve T cells in vivo [60]. However, a strong TCR signal can overcome the stress induced by the KDELR mutation, maintain naïve cells, and restore the ability of the cells to proliferate [61]. ATF4, which is upregulated by phosphorylated eIF2α, plays a role in proliferation as well. ATF4 deficient murine T cells have reduced proliferation [62], while Xbp-1 and CHOP appear to play a role in T cell quiescence. T cells in Schlafen-2 mutant mice have chronic ER stress, with a greater proportion of T cells adopting an activated state and T cell death, and deletion of Xbp-1 or CHOP increases viability and partially corrects for developmental defects [63,64].

The GTPase domain of the immune-associated nucleotide-binding protein 5 (Gimap5, also known as IAN5) regulates natural killer T cell (NKT) functions and NKT development and peripheral T cell survival, proliferation, and Ca^2+^ homeostasis in mouse and rat models, as loss of Gimap5 negatively effects these processes [65,66,67,68]. Moreover, Gimap5 is upregulated in single positive (SP) CD4^+^ and CD8^+^ thymocytes, and knocking down Gimap5 in fetal mouse thymocytes inhibits the development of double positive and CD4^+^ SP T cells [69]. Gimap5 regulates T cell homeostasis and proliferation in peripheral T cells through inhibition of glycogen synthase kinase 3β (GSK3β), a UPR protein found to block CHOP mediated apoptosis [68,70,71].

### 2.3. CD4^+^ T Helper Cells

CD4^+^ T cells are involved in many aspects of the immune response and it is well established that CD4^+^ T cell differentiation is dependent on the strength and length of the TCR signal and the microenvironment. Th1 cells, identified by production of IFNγ, are involved in intracellular responses; Th2 cells, identified by production of IL-4, are involved in extracellular responses; Th17 cells, identified by the production of IL-17, are involved in inflammatory responses; Th9 cells, identified by the production of IL-9, are involved in responses to parasites; and T regulatory cells are involved in regulating immune functions [72]. Recently, the UPR and ER stress have been shown to play a role in T helper differentiation and effector functions.

The IRE1α/Xbp-1 pathway appears to play a role in promoting Th2 differentiation and effector functions. Xbp-1 promotes Th2 differentiation and activation [73], and induction of Th2 and type 2 innate lymphoid cell (ILC2) effector functions in mice are dependent on IRE1α and Xbp1s [74,75]. CD4^+^ T cells from IRE1α mutant mice have reduced IL-4 production, *Xbp1s* mRNA, and *IL-4* mRNA stability in vitro upon activation and Th2 differentiation [55]. Activation of IRE1α mutant T cells in the presence of 4μ8c, a small molecule inhibitor of IRE1α RNase function, leads to a further reduction in *Xbp1s* mRNA levels and inhibition of Th2 cytokines IL-4, IL-5, and IL-13. Moreover, treatment of human and mouse T cells activated in the presence of 4μ8c, a small molecule inhibitor of IRE1α RNase function, leads to reduced secretion of IL-4, IL-5, and IL-13 [55,76,77]. Human Th2 cells differentiated in the presence of 4μ8c have reduced IL-4 production; however, a study measuring IL-4 production in mouse cells treated under similar conditions found IL-4 secretion was inhibited, while cells maintained the ability to produce IL-4 as detected by flow cytometry [76,77]. The discrepancy between these studies may be due to differences in species. Interestingly, while inhibition of UPR reduces Th2 effector function, cellular stress that promotes ER stress is reported to also reduce Th2 cytokine production [78]. This indicates that there may be a “Goldilocks Zone” with regards to ER stress, too little or too much leads to effector functions being inhibited.

UPR appears to regulate Th2 cytokines differently in Th2 cells with an established phenotype. Treatment of established human Th2 cells and a mouse Th2 cell line, D10.G4.1, with 4μ8c leads to a reduction of IL-5 secretion, but not IL-4 [77]. Moreover, IL-5 is still produced in the mouse cells treated with 4μ8c, indicating that inhibition of IRE1α in established Th2 cells results in loss of IL-5 secretion, but not production. Interestingly, strength of signal appears to play a role in the regulation of Th2 cytokines by UPR, as there was a modest reduction in IL-13 production in mouse cells stimulated with strong agonists that mimic a T cell signal, Phorbol myristate acetate and ionomycin, but not plate-bound antibody. In addition, IL-4 translation appears to be regulated by eIF2α in primed Th2 cells; restimulation of Th2 cells results in dephosphorylation of eIF2α and translation and production of IL-4 [79].

While there is considerable evidence for a role for UPR in Th2 effector functions and cytokine regulation, UPR appears to play a lesser role in Th1 cells. Induction of cell stress via inhibition of glucose metabolism or osmotic stress, events that activate ISR, results in reduced IFNγ production in mouse Th1 cells [78]. While ATF4 is not required for T cell development in the thymus, it appears to be necessary for Th1 effector functions [38,62]. Using a fetal liver cell transfer (FTL) mouse model, Yang et al. showed that loss of ATF4 leads to diminished Th1 effector function in high and low oxidizing environments [62]. Moreover, IFNγ was reduced in isolated splenocytes from *ATF4^-/-^* FTL mouse cells using the experimental autoimmune encephalomyelitis (EAE) and keyhole limpet hemocyanin (KHL) models. Induction of ATF4 in T cells undergoing activation in a high oxidizing environment or under amino acid deprivation appears to be induced in part by general control nonderepressible proteins 2 (GCN2), a kinase that phosphorylates eIF2α in response to proteasome inhibition and nutritional deprivation as part of the ISR [62,80]. ATF4 regulates mechanistic target of rapamycin complex 1 (mTORC1), as inhibition of ATF4 leads to reduced mTORC1 pathway activation [62]. The mTORC1 pathway promotes Th1 differentiation [81,82]. However, IRE1α and PERK do not appear to play a direct role in Th1 effector functions, as loss or inhibition of these molecules in Th1 cells or cells being differentiated under Th1 conditions does not block Th1 effector functions and profile [55,76,77,83]. This indicates that PERK is not important for promoting ATF4 in Th1 cells, and that ATF4 may be activated by one of the many other pathways that are explained in the introduction that promote eIF2α phosphorylation.

Loss of ATF4 led to a modest reduction in IL-17 production in vitro under high oxidizing conditions, presumably due to a reduction in the activation of mTORC1 [62]. The mTORC1 pathway is implicated in regulating Th17 cells and IL-17 production, and leptin induces mTOR, a component of mTORC1 [81,84,85]. Leptin activates the mTOR via the phospoinositide-3-kinase/Protein kinase B (also known as Akt) pathway [85,86,87]. Interestingly, leptin, which can induce Th17 and Th2 cell differentiation and effector functions, is found to activate Th2 differentiation via activation of a signaling pathway that involves mTOR promoting IRE1α activation and *Xbp1s* [74,88,89,90]. Moreover, leptin promotes the survival of autoreactive T cells, including IL-17 and IFNγ producing cells and the ability of the cells to induce EAE via activation of mTOR [91]. ATF4 deficient mice have reduced clinical disease in an EAE model [62]. It may be that targeting ATF4 will be of benefit in the treatment of autoimmunity due to its influence on mTOR and IRE1 [11].

CHOP has been implicated in the regulation of IL-17 [92]. However, a recently published study found *CHOP*^−/−^ mice had normal Th17 differentiation [78]. Brucklacher-Waldert et al. found that cellular stressors such as hypoxia, changed ionic pressure, and reduced glucose metabolism promote activation of the UPR via Xbp-1 and enhanced Th17 differentiation, albeit to different degrees, while inhibition of cellular stress via treatment with Tauroursodeoxycholic acid (TUDCA), a water-soluble bile salt known to block ER stress response, delayed the onset of Th17 mediated autoimmunity in a mouse model of multiple sclerosis. They also found treating cells with activators of ER stress promoted Th17 effector functions. Moreover, in the same study, onset of disease symptoms was shown to be delayed in mice with Xbp-1 deficient lymphocytes. The data in this study corroborated with other studies that found that hypoxia and disruption of osmotic pressure induced Th17 effector functions [93,94,95,96,97]. However, prolonged hypoxia without reoxgynation results in reduced IL-17, while culturing cells under hypoxic conditions with normoxic exposure (5% oxygen) leads to elevated IL-17 [93,98]. In contrast, a 2011 study by Shi et al. found that blocking glycolysis using a similar method to the Brucklacher-Waldert study resulted in reduced Th17 effector functions and promotion of Treg differentiation in vitro [94]. Differences in results between these two studies may be explained by seeding densities and/or media which can change the microenvironment and the presence of environmental stressors. However, Shi et al. also found that treatment of mice with an inhibitor of glycolysis reduced EAE. A recent study looking at the role of glycolysis in Th17 and Treg cell functions using human peripheral blood mononuclear cells (PBMCs) found inhibiting glycolysis blocks Th17 differentiation and reduced IL-17 production in memory T cells and the survival of memory T cells [99]. This study also found that while Treg cells utilize glycolysis, inhibiting glycolysis in memory Tregs does not block their ability to function. Future studies will have to be performed to better define the role of glycolysis and UPR in Th17 effector functions. Interestingly, while PERK and IRE1α are not required for Th17 differentiation, as their inhibition does not preclude Th17 differentiation [55,83], inhibition of PERK and IRE1α mitigates the effects of cellular stress on Th17 differentiation caused by reduced glucose metabolism or hypoglycemia and changed ionic pressure, respectively [78]. This indicates that different arms of UPR are important for promoting Th17 effector functions dependent on various environmental stressors.

### 2.4. T Regulatory Cells

ER stress is implicated in the regulation of regulatory T cells. CD4^+^ and CD8^+^ human T regulatory (Treg) clones had elevated IL-10 cytokine production when treated with thapsigargin, an activator of ER stress and UPR, in an eIF2α phosphorylation dependent manner [100]. Loss of ATF4 led to a modest increase in *FOXP3* mRNA expression in mouse CD4^+^ cells differentiated under T regulatory conditions in a high oxidizing environment [62]. In addition, loss of molecular chaperone GP96 led to instability in mouse Treg cells resulting in loss of FOXP3 expression and promotion of IFNγ production [101]. Interestingly, inhibition of GP96 led to a modest increase in ATF4 in hepatoma cells [102], causing one to ask if the loss of FOXP3 and promotion of IFNγ in GP96 deficient Tregs is due to elevated ATF4. Indeed, ATF4 promotes Th1 responses [62]. GP96 also regulated the availability of TGF-β on the surface of T regulatory cells [101]. This indicates that GP96 plays roles in T cell activation and plasticity.

### 2.5. CD8 T Cells

CD8^+^ T cells, known as cytotoxic T cells, play an important role in immune responses to cancer and internal pathogens. ER stress plays an important role in CD8^+^ T cell differentiation and gain of effector functions. Infection of mice with Lymphocytic choriomeningitis virus resulted in upregulation of spliced *Xbp-1*, and Xbp-1 enhanced differentiation of CD8^+^ T cells [103]. ER stress chaperone GRP78 also appears to play a role in the regulation of granzyme B in CD8^+^ T cells and CD8^+^ Intraepithelial lymphocytes, as GRP78 heterozygous CD8^+^ T cells had reduced granzyme B secretion and cytotoxicity [56]. This granzyme B deficiency, found in GRP78 heterozygous mouse T cells, was due to a reduction in IL-2 mediated proliferation, as exogenous IL-2 helped to partially restore granzyme B expression.

### 2.6. Invariant Natural Killer T Cells 

Invariant natural killer T cells (iNKT) cells are an innate-like T cell subset that recognizes glycolipid antigens presented by a non-classical MHC molecule, CD1d and express natural killer cell markers on their surface. iNKT cells rapidly produce cytokines following activation, and like other T cells, can be further divided into effector lineages based on their cytokine profiles [104]. iNKT1 cells produce IFNγ and CCL5, iNKT2 cells produce IL-4 and IL-13, and iNKT17 cells produce IL-17, IL-4, and IL-13. Moreover, these subsets of cells appear to play important roles in anti-tumor immunity and promoting autoimmunity and asthma [104,105,106,107,108]. The IRE1α/Xbp-1 pathway is active within iNKT1 and iNKT17 lineages, and UPR is upregulated upon TCR stimulation [109]. Interestingly, IRE1α deficient iNKT1 and iNKT17 cells experience globally impaired cytokine production due to reduced mRNA stability. This is similar to how IL-4 is regulated in primary CD4^+^ T cells isolated from IRE1α deficient mice [55]. In addition, IRE1α is implicated in regulating *GATA3* and *T-bet* mRNA in iNKT cells, as palmitic acid-mediated ER stress leads to degradation of *GATA3* and *T-bet* mRNA via RIDD, which results in reduced IL-4 and IFNγ [110]. Moreover, these transcription factors are important for iNKT1 and iNKT17 effector functions [111].

## 3. UPR, T Cells, and Disease

ER stress has been implicated in neurological diseases, diabetes, cancer, and various inflammatory disorders. Moreover, ER stress and the activation of the UPR influences the differentiation and effector functions of T cells and, in turn, manipulating UPR in T cells may be a way to combat a number of diseases and disorders. In the following sections, we speculate on how UPR may be manipulated in T cells to combat disease.

### 3.1. Cancer

The enhanced proliferation found in cancer cells leads to a nutrient deprived environment, hypoxia, and an increased influx of unfolded protein in the ER, all of which activate UPR [112,113,114,115]. Moreover, tumor cells alter their surroundings to create a supportive and immunosuppressive microenvironment. T cells play an important role in cancer responses; however, T cells found in the tumor microenvironment experience metabolic constraints which can affect T cell effector function [116,117,118]. Interaction of T cells with tumors results in reduced function and death for T cells due to the secretion of soluble factors and through cell to cell interactions [119,120,121,122]. Manipulation of UPR appears to be an avenue for the maintenance of T cell function and health in tumor microenvironments.

CD4^+^ T cells play an important role in tumor immunity. CD4^+^ T cell death is induced by tumor supernatants in a manner that induces ER stress-mediated apoptosis [123]. IRE1α was found to be activated in T cells found in the ovarian cancer microenvironment, leading to decreased IFNγ, suppressed mitochondrial activity in CD4^+^ T cells, and increased infiltration of T cells in patient tumors. Inhibiting the IRE1α-Xbp-1 pathway activation in human T cells enhanced mitochondrial respiration. In addition, mice with Xbp-1 deficient T cells mounted a tumor response, experienced a delay in malignancy, and had enhanced survival in an ovarian cancer model [122,124]. This work is of particular interest as Xbp-1 appears to promote CD8^+^ T cell effector functions in response to infection [103]. It may be that the cell microenvironment determines whether the IRE1α-Xbp-1 pathway will promote or inhibit T effector functions.

There is evidence that the UPR can be manipulated to promote the clearance of tumors. Ca^2+^ mobilization is important for T cell activation, and blocking inositol triphosphate receptor, a Ca^2+^ channel, in the ER in vivo promotes tumor responses in T cells [57]. Ca^2+^ accumulation in the ER is regulated by sarco/endoplasmic reticulum Ca^2+^ ATPase (SERCA) pumps, and inhibition of SERCA induces ER stress [125,126]. It may be that nifetepimine can be used to inhibit tumors and promote T cell responses. Tumor induced SERCA3 upregulation in T cells resulted in apoptosis via activation of the UPR, and inhibition of this process by treatment with nifetepimine resulted in T cell survival and downregulation of SERCA3 [123]. This is particularly interesting because nifetepimine promoted apoptosis in triple negative breast carcinoma cells due to inhibition of GRP78 upregulation, and it is thought that tumors survive in part due to activation of the UPR [127,128]. Genetic ablation of the ER stress molecule GP96 resulted in reduced Ca^2+^ mobilization in mouse T cells and reduced glucose metabolism [57]. GP96 expression is associated with a number of cancers [129,130]. Therefore, due to the importance of GP96 in promoting cancer, any therapies that promote GP96 in T cells will have to be carefully targeted. GP96 is also upregulated in primary lymphomas caused by Marek’s disease virus, a pathology found in chickens that is used as a model for lymphomas that express Hodgkin’s disease antigen CD30 [131,132,133]. ATF6 promotes Xbp-1 and GP96 [14,58] expression, and ATF6 expression correlates with poor prognosis in a number of cancers [134,135]. However, it remains to be determined if ATF6 can directly influence T cell function in tumor immunity.

ATF4 is important for metabolism in T cells undergoing activation and regulates T helper effector functions [62], making ATF4 a potential target for cancer treatment. Indeed, there is interest in targeting ATF4 for tumor therapy, as ATF4 is overexpressed in many cancers and appears to promote the survival of tumors [136,137,138,139]. Loss of ATF4 in mice led to a reduction in Th1 cells when differentiated in vitro and increased FOXP3 expression in T cell cultures under high oxidizing conditions [62]. Th1 cells promote the clearance of tumors in many different cancer models [140,141,142]. However, any therapies that inhibit ATF4 in T cells will need to be targeted and narrow in scope, as a global loss of AFT4 leads to increased Th17 responses in vivo due to effects on non-T cells [62]. Alterations in the balance of Th17 and Treg cells are reported in a number of cancers [143,144]. Moreover, Th17 and Treg cells are both implicated in promoting and inhibiting anti-tumor activities [72,145,146]. ATF4 presents a potential target for helping maintain the balance between Th17 and Treg cells in tumor immunity.

UPR is also a potential target for the treatment of leukemias. Ubiquitin fusion degradation 1 (UFD1) is a component of the ERAD complex and promotes the elimination of misfolded proteins [147]. UFD1 inactivation in human T- acute lymphoblastic leukemia (T-ALL) induces apoptosis via activation of PERK [148]. There are a number of proteasome inhibitors available for treatment of leukemia. The proteasome inhibitor bortezomib induces apoptosis via activation of ER stress responses, and in combination with casein kinase 2 inhibitor CX-4945, which also induces apoptosis via UPR, these two drugs functioned to induce apoptosis of various T-ALL and B cell acute lymphoblastic leukemia (B-ALL) lines and primary lymphoblasts from T and B-ALL patients [149]. The drugs inhibited GRP78 activation, while promoting IRE1, CHOP, PERK, and eIF2α phosphorylation. Treatment of Jurkat cells, a T-ALL derived cell line, with chemotherapeutic agent selenite, led to activation of ATF4 and CHOP and promotion of cell death [150]. Moreover, farnesol, a naturally occurring isoprenoid alcohol, induced apoptosis and blocked proliferation of human T lymphoblastic leukemia Mot4 cells, and this correlated with upregulation of CHOP and other UPR associated molecules [151]. Interestingly, just as with the combination of CX-4945 and bortezomib, farnesol reduced GRP78 expression. GRP78 when overexpressed promotes chemoresistance in cancer cells [152,153], and GRP78 levels appear to be predictive of chemoresponsiveness [154,155,156]. Therefore, targeting GRP78 and other UPR molecules in T cell leukemias is another potential avenue for treatment.

### 3.2. Asthma

Asthma is a lung disease that inflames and constricts the airway, leading to difficulty breathing. It has a number of distinct clinical phenotypes caused by interactions of different leukocytes, epithelial cells, and stromal cells, and while asthma is classically thought of as a type 2 immune response disease mediated by cytokines IL-4, IL-5, and IL-13, other T cell types such as Th1, Th17, Treg, and NKT cells are implicated in asthma pathology [107,157,158,159,160,161]. Moreover, T cells are known to retain plasticity and are influenced by their surroundings, allowing them to manifest attributes typically assigned to other subsets [162,163,164,165]. While the majority of cases can be controlled using standard treatments, a subset of asthmatic patients is unable to control the disease.

The IL-5-producing highly pathogenic memory Th2 cells, also termed memory type pathogenic Th2 cells (Tpath2), are important in inducing pathogenesis in allergic airway inflammation and atopic dermatitis [166,167]. IL-4 produced by these cells promotes Th2 differentiation and IgE isotype switching in B cells; IL-5 produced by these cells recruits eosinophils, leading to eosinophilia; and IL-13 produced by these cells targets the airway epithelial cells to induce airway hyperresponsiveness. IRE1 regulates Th2 cytokine production and differentiation in vitro and in vivo [55,73,75,76,77]; moreover, inhibiting IRE1 in established Th2 cells leads to a block in IL-5 secretion, indicating that UPR could be used to target Tpath2 cells

Obesity is a major risk factor for asthma, and elevated leptin, an adipokine mainly produced by adipocytes, correlates with allergic asthma [168,169]. While obesity and a high fat diet appear to promote exacerbated lung disease, there is some debate over how T cells are affected by leptin. It is clear that leptin is critical for T helper responses in vitro and in vivo [74,170,171]. There is evidence that leptin can upregulate cytokines such as IFNγ and IL-17 in asthma and can both promote and inhibit Th2 effector functions in vitro and in type 2 diseases such as asthma and allergic rhinitis [170,171,172,173,174,175]. This variation may be due to the concentration of leptin present. Leptin can affect nearby cells that influence T helper effector functions [176,177,178]. Changes in T cell subpopulations occur in the adipose tissue and blood of obese individuals overtime; moreover, there is evidence that metabolic abnormality can influence T helper effector cytokine profile [179,180,181].

Leptin, either applied exogenously or upregulated via a high fat diet in mice, promotes type 2 lymphocyte responses and airway hyperresponsiveness, and there is evidence that this requires activation of the IRE1α/Xbp-1 pathway in T cells [74,75,172,182]. Feeding mice a high fat diet increased Xbp1s expression in splenocytes and elevated Th2 responses and airway hyperresponsiveness [75]. Knocking down *Xbp1s* led to reduced Th2 responses in T cells stimulated with leptin and promoted death.

Leptin and obesity also promote Th17 differentiation, and Th17 cells are elevated in the visceral adipose tissues and peripheral blood of obese individuals [88,179,183,184]. A study by Silva et al. found obesity in mice leads to elevated IFNγ, IL-4, and IL-17 in lung tissue homogenates [172]. Leptin also appears to promote airway hyperresponsiveness in obese mice in a Th17 dependent manner [185]. While Zheng et al. did not explore whether IL-17 was elevated in their obese mice experiencing airway allergic responses, they do find neutrophil infiltration increased in the lung, and Th17 cells play an important role in recruiting neutrophils [75,186]. Treating obese mice with celastrol, a drug known to promote apoptosis in cancer cells via activation of IRE1, ATF4, and CHOP, reduces airway hyperresponsiveness and IL-17 production [185,187,188,189]. These studies indicate that targeting UPR in obesity mediated asthma may prove beneficial for treatment.

Loss of Gimap5 promoted pathogenic Th2 and Th17 cells in humans and mice and led to an exacerbated lung allergic inflammation and airway hyperresponsiveness in mice [190], and Gimap5 is implicated in regulating CHOP activity [68,69]. This does not indicate a direct role of CHOP in airway hyperresponsiveness; however, arsenic trioxide treatment reduced airway hyperresponsiveness, lung inflammation, and reduced Th17 cells in mouse models of asthma [191,192,193,194]. This treatment promoted T cell apoptosis, and knocking down CHOP in T cells provided some protection to T cells from arsenic trioxide treatment [192]. In addition, celastrol, which reduces Th17 associated airway hyperresponsiveness caused by obesity in mice, is known to promote upregulation of CHOP and apoptosis and downregulates GSK3β [185,188]. GSK3β, which is found downstream of Gimap5, inhibits CHOP induced apoptosis [70,187,188]. These studies hint at an importance of CHOP in controlling airway hyperresponsiveness and lung inflammation; however, future studies will have to explore a role for CHOP in the pathology of asthma or allergy and whether it is protective or harmful.

ER stress and UPR are implicated in the regulation of allergic responses and in T cells involved in these diseases. ER stress also appears to mediate mast cells and eosinophil responses [32,195]. Moreover, treatment of mice with 4μ8c, an inhibitor of IRE1α RNase activity, suppressed passive cutaneous anaphylaxis, a disease mediated by tissue mast cells [195]. Therefore, targeting ER stress in models of asthma, as well as other type 2 diseases, may prove to be protective due to effects on T helper cells and other immune cells involved in these responses.

### 3.3. Rheumatic Diseases

T cells are involved in the pathology of a number of rheumatic diseases. While the manifestations of systemic lupus erythematosus (SLE) are mediated by autoantibodies against nuclear antigens and other self-proteins, dysregulation of T cell function and population ratios helps promote disease [196,197]. Moreover, pathology is thought to develop in part due to enhanced T cell apoptosis [198,199]. Because UPR is important in T cell effector functions, as explained earlier in this text, targeting UPR associated molecules in T cells may help protect against SLE. T cells in SLE patients have altered expression of adhesion markers, cell signaling pathways, and TCR subunits [196]. Moreover, SLE disrupts T lymphocyte homeostasis, and UPR helps regulate T cell homeostasis. In addition, there is evidence for oxidative stress in T cell dysfunction in SLE, and oxidative stress induces genes involved in UPR [4,200]. A 2014 study found *CHOP*, *IRE1*, and *PERK* to be downregulated in PBMCs of SLE patients, while total *Xbp-1* and *Xbp-1s* was upregulated [201]. T cells make up between 50–75% of the total PBMCs in normal individuals [202], so one assumes that the elevated gene expression is due in part to the T cells present in SLE individuals. Moreover, T lymphocytes from SLE patients are more susceptible to ER stress induced apoptosis [203]. There is also evidence that UPR contributes to T cell responses in the development of arthritis. Dietary palmitic acid, an inducer of ER stress and RIDD in NKT cells, and tunicamycin, an activator of ER stress and UPR, inhibit IL-4 and IFNγ in a mouse model of arthritis and leading to reduced antibody induced-joint inflammation [110]. Because UPR appears to play an important role in NKT cell effector responses, targeting UPR in these cells may protect against joint inflammation.

Antibodies to GRP78, GRP94, and Calnexin, an ER resident chaperone involved in protein folding, are found in the sera of SLE and rheumatoid arthritis (RA) [204]. In particular, GRP78 is a major autoantigen for human T cells in patients [205,206]. GRP78 is upregulated in the synovial sections of RA patients, and T cells reactive to GRP78 are found in RA patients [207]. Preimmunization of rats and mice with GRP78 stops the development of arthritis [205]. Moreover, stimulation of human PBMCs and CD8 T cells with GRP78 results in production of IL-10 and the development of a regulatory phenotype [206,208]. Treatment of mice with GRP78 intravenously or subcutaneously during the active phase of the collagen induced arthritis model reduced disease severity and led to increased IL-4, IL-5, and IL-10 (associated with dampening immune responses) production [209]. Moreover, this improvement was dependent in part on cytokine IL-4, as GRP78 treatment was not able to lessen disease severity in IL-4^−/−^ mice immunized with GRP78. Interestingly, high levels of GRP78 did not improve severity in mice. Peripheral T cells in RA patients proliferate to GRP78 in a HLA-DR dependent manner unlike healthy individuals [207,208]. Shoda et al. show through using human PBMCs from individuals with RA that GRP78 derived epitopes are differently recognized by effector T cells and T regulatory cells. The epitope that induced the greatest response (immunopromoting epitope) activated effector T cells from RA patients, but not healthy individuals, as evidenced by elevated IFNγ and IL-17 and increased proliferation, while an epitope that produced a weaker T cell response (immunoregulatory epitope) lead PBMCs to produce cytokine IL-10 [210]. In addition, stimulating PBMCs with the immunopromoting epitope in the presence of the immunoregulatory epitope reduced IL-17 and IFNγ production, and introducing the immunoregulatory peptide orally in a collagen induced arthritis model led to increased IL-10 and T regulatory cells. It may be that the effector response is activated at higher concentrations of GRP78. Weber et al. found antibodies against GRP78, GRP94, and calnexin in RA individuals early into disease progression [204]. These molecules may also play a role in both pathogenicity and in protection, with higher concentrations of GRP78 promoting disease progression in mice [206,209]. In addition, T cells from SLE patients also proliferate in response to GRP78, albeit to a lesser degree when compared to T cells from RA individuals [210]. These studies indicate that UPR could be targeted to treat RA and potentially SLE.

## 4. Conclusions

**Figure 3:**
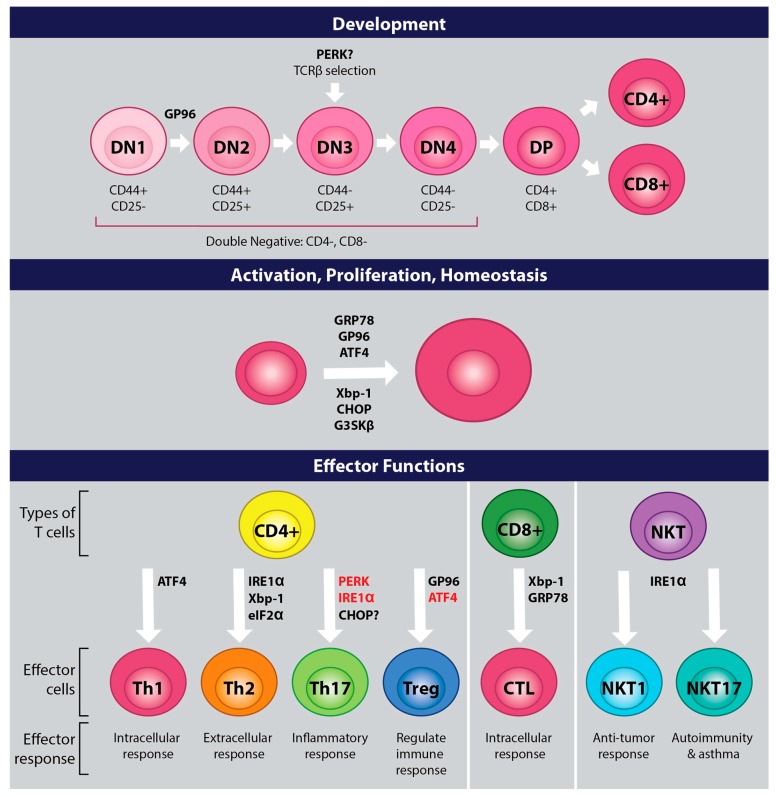
Roles for UPR molecules in T cells. UPR associated proteins play important roles at all stages in the life of a T cell. Proteins in black appear to play a direct role in T cells, while proteins in red promote effector functions when the T cell is placed in a less than optimal environment (i.e., reduced glucose metabolism, amino acid deprivation, oxidizing environment, etc.). Proteins with a question mark may play a role, but further studies need to be embarked upon.

Changes that occur in T cells related to development, proliferation, activation, and differentiation, lead to upregulation of ER stress and UPR. UPR is critical for the survival of cells due to its role in maintaining ER stress homeostasis. Much work remains with regards to exploring how UPR and ER stress control T cell immunity; however, the studies highlighted in this review suggest that UPR is critical for T cell development and immune functions and that UPR could play an important role in regulating diseases mediated by T cells (Figure 3). Future work is required to determine the efficacy of treatments that modulate UPR on disease induction and progression.

## Abbreviations

ER	Endoplasmic Reticulum
SRP	signal recognition protein
ERAD	endoplasmic reticulum-associated degradation
RIDD	regulated IRE1-dependent decay
UPR	unfolded protein response
TRPV1	transient receptor potential cation channel subfamily V member 1
PERK	protein kinase RNA-like endoplasmic reticulum kinase
ATF6	activating transcription factor 6
IRE1	and inositol-requiring enzyme 1
eIF2α	eukaryotic translation initiation factor alpha
Rpl22	ribosomal protein 22
ATF4	activating transcription factor 4
eIF4G	eukaryotic initiation factor 4 G
eIF4F	eukaryotic initiator factor 4 F
CHOP	C/EBP homologous protein
XBP-1	X box binding protein 1
GP96	glucose-regulated protein
HSC	hematopoietic stem cells
ISR	Integrated stress responses
TCR	T cell receptor
GRP78	Glucose-Regulated Protein 78
Lck	lymphocyte kinase
NK	natural killer
NKT	natural killer T cell
SP	single positive
DP	double positive
DN	double negative
KDELR	KDEL receptor 1
SERCA	sarco/endoplasmic reticulum Ca^2+^ ATPase
FTL	fetal liver transfer
mTORC1	mechanistic target of rapamycin complex 1
GCN2	general control nonderepressible proteins 2
EAE	Experimental autoimmune encephalomyelitis
ILC2	type 2 innate lymphoid cells
KHL	keyhole limpet hemocyanin
iNKT	Invariant natural killer T cells
T-ALL	T- acute lymphoblastic leukemia
UFD1	Ubiquitin fusion degradation 1
SLE	systemic lupus erythematosus
PBMCs	peripheral blood mononuclear cells
RA	rheumatoid arthritis
GSK3β	glycogen synthase kinase 3β
Gimap5	GTPase domain of the immune-associated nucleotide-binding protein 5
Tpath2	memory type pathogenic Th2 cells
